# Biological Treatments for Temporomandibular Joint Disc Disorders: Strategies in Tissue Engineering

**DOI:** 10.3390/biom11070933

**Published:** 2021-06-23

**Authors:** Daniela Trindade, Rachel Cordeiro, Henrique Cardoso José, David Faustino Ângelo, Nuno Alves, Carla Moura

**Affiliations:** 1 Centre for Rapid and Sustainable Product Development, Polytechnic of Leiria, 2430-028 Marinha Grande, Portugal; daniela.trindade@ipleiria.pt (D.T.); rachel.s.cordeiro@ipleiria.pt (R.C.); david.angelo@ipface.pt (D.F.Â.); 2 Instituto Português da Face, 1050-227 Lisboa, Portugal; henrique.cardoso@ipface.pt; 3 Faculdade de Medicina, Universidade de Lisboa, 1649-028 Lisboa, Portugal

**Keywords:** temporomandibular joint disc, fibrocartilage, disc dysfunctions, tissue engineering, decellularization

## Abstract

The temporomandibular joint (TMJ) is an important structure for the masticatory system and the pathologies associated with it affect a large part of the population and impair people’s lifestyle. It comprises an articular disc, that presents low regeneration capacities and the existing clinical options for repairing it are not effective. This way, it is imperative to achieve a permanent solution to guarantee a good quality of life for people who suffer from these pathologies. Complete knowledge of the unique characteristics of the disc will make it easier to achieve a successful tissue engineering (TE) construct. Thus, the search for an effective, safe and lasting solution has already started, including materials that replace the disc, is currently growing. The search for a solution based on TE approaches, which involve regenerating the disc. The present work revises the TMJ disc characteristics and its associated diseases. The different materials used for a total disc replacement are presented, highlighting the TE area. A special focus on future trends in the field and part of the solution for the TMJ problems described in this review will involve the development of a promising engineered disc approach through the use of decellularized extracellular matrices.

## 1. Introduction

The temporomandibular joint (TMJ) is a synovial joint between the temporal bone and the mandibular condyle, located bilaterally in the face. This joint is composed of bony articular surfaces, articular disc, fibrous capsule and synovial membrane, ligaments and muscles. It is responsible for basic functions, such as talking, chewing, swallowing, eating, yawning, smiling, laughing, screaming and kissing [1]. The articular disc is a crucial element present in the TMJ, as it softens and absorbs shocks between the articular structures. It separates the joint cavity in the upper and lower compartments, and it is surrounded by attachments that sustain its position [2].

This joint is surrounded by a synovial capsule, whose main function is to produce synovial fluid. This fluid plays a major role in joint lubrication and acts as a medium for nutrient and waste exchanges. As part of the joint, the disc is an avascular structure that relies heavily on nutrients and oxygen from the synovial fluid to survive [3]. Lubrication plays an essential role in the rotational and translational movements of the TMJ. These two types of movements take place between the condyle and the articular disc and between the mandibular fossa and the articular disc, respectively [4], it is therefore considered a ginglymoarthrodial joint [5].

Temporomandibular joint disorder (TMD) is a type of musculoskeletal pain that affects the orofacial region, like masticatory muscles, temporomandibular joint and other surrounding structures [6]. Chronic musculoskeletal pain refers to a persistent pain, felt for more than 3 months, arising in bones, joints, and tissues [7]. Statistics for 2002 indicate that, in Europe, about 95 million adults report having musculoskeletal pain associated with arthritis or rheumatism, corresponding to over 3 million Portuguese [8].

Symptoms of TMD include headache, neuralgia, pain and discomfort, clicking sounds and muscle spasms [9,10], affecting patients’ quality of life and daily/work functions [11]. TMD is considered the second most common musculoskeletal disorder affecting the general population in 5–12% [12].

In a study conducted by AlShaban and Gul Abdul Waheed (2018), 41 patients out of 100 revealed the presence of TMD. Of this percentage, the clicking sound appeared as the symptom with the highest prevalence, affecting 89% of the patients. Of these 89% patients, clicking sound was 32% from the right side, 24% from the left side, 32% on both sides and the remaining 12% were absent [13].

Just as disorders affect the TMJ and surrounding tissue, clinical options also solve problems in these tissues, including the disc. These treatments can vary according to the stage and severity. They can be classified into three categories: (i) non-invasive; (ii) minimally invasive; or (iii) invasive. Non-invasive procedures are the first option of therapy for TMD patients and include medications, such as anti-inflammatory drugs and muscle relaxants, physical therapy and acupuncture. Minimally invasive treatments can be divided into intra-articular injections, which involve the injection of medications and/or sodium hyaluronate. Arthrocentesis and arthroscopy are also in this category and are used to lubricate the joint and eventually reposition the articular disc. Invasive treatments are the last option to be performed and include open joint surgery, where there can be a discopexy (disc repositioning), discectomy (total removal of the articular disc), condylectomy (excision of the condyle), or a total joint replacement with compatible materials [14,15,16,17,18]. Recent strategies have improved the surgical cosmesis of the open surgery incision [19,20].

Regarding TMJ disc clinical possibilities, minimally invasive procedures do not restore damaged disc, and discectomy leads to condylar remodeling [21]. Considering this, the demand for a possible material to replace the disc has been investigated. The first materials used for TMJ disc replacement after discectomy were Silicone rubber and Proplast-Teflon [22,23]. After that, the field of Tissue Engineering (TE) became active to find a solution for disc pathologies. Through the use of natural or synthetic materials, it is possible to produce bioengineered scaffolds for the repair of the entire or only a portion of the disc [24]. Despite the search for a promising bioengineered scaffold-based TE strategy, the need for a successful TMJ disc remains. The use of decellularized tissue could be a potential substitute to synthetic materials since its three-dimensional (3D) architecture and biological composition are the same as the native one [25].

In this review, the different materials used for a total disc replacement are discussed, as well as the biomaterials used for this purpose in the area of tissue engineering, with emphasis on future trends in this field, such as the use of decellularized extracellular matrices.

## 2. Temporomandibular Joint Disc Characterization

The TMJ movement is obtained through an articulation of the mandibular condyle with the temporal bone, with the interposition of an articular disc between those structures [26,27,28]. Throughout the normal masticatory function, bone-to-bone contact is prevented by the mandibular condyle-disc complex, which slides anteriorly [29].

**Figure 1:**
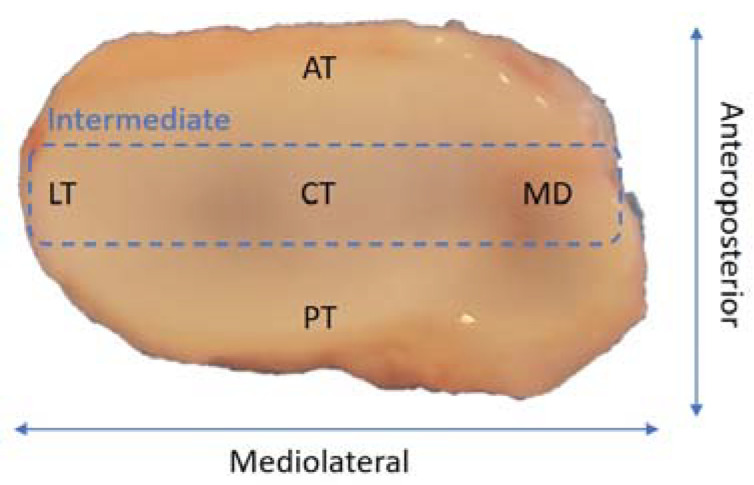
Different regions of the temporomandibular disc: anterior (AT), posterior (PT), lateral (LT), central (CT) and medial (MD).

The articular disc is an avascular and non-innervated tissue composed of fibrocartilage with viscoelastic consistency. It has a biconcave shape, thinner in the central region and thicker in the periphery, approximately 1–2 mm. The TMJ disc can be divided into three regions: anterior, intermediate and posterior. The intermediate zone can also be subdivided into 3 regions: lateral, central and medial (Figure 1) [26,30,31,32].

An important attachment, called the retrodiscal tissue, is attached posteriorly to the disc controlling its position during jaw function [33].

The disc is composed by a mixture of chondrocytes and fibroblasts (30% and 70%, respectively). It is mainly constituted by type I collagen, but it also presents type II collagen, mostly found in the intermediate zone. It presents approximately 74.5% water content of by wet weight and it is further composed of proteoglycans and glycosaminoglycans (GAGs) and elastic fibers [3,26,34,35,36,37]. The disc is an essential element in normal TMJ with the following functions: (i) provides a smooth interface between the condyle and the mandibular fossa; (ii) load-bearing and support forces (e.g., compression, tension and shear forces); and (iii) lubricates the surrounding surfaces for the different range of motions [21,35,38]

The interaction of the different biochemical components provides the mechanical properties of the disc [39]. Disc morphology is restored with the help of elastin fibers and GAGs are related to the compressive strength of the disc [40]. Collagen fibers present an anisotropic variation and are highly correlated with the tensile properties of the disc [41]. The disc is softer under compression when compared to tensile forces, exhibiting a compressive modulus between 0.1 and 10 MPa [42].

## 3. Temporomandibular Joint Disc Disorders

A subset of disc-related TMD involves internal derangement (ID) or disc displacement (DD), disc thinning and disc perforation. As will be explained in Section 3.2, disc thinning and perforation could be related to ID, where these events represent 5–15% of ID patients. However, it may not be related to this problem [5,21]. Biomechanical unbalance or extreme loading can also lead to damage in the articular disc [43].

### 3.1. Disc Displacement

Normal jaw function may be affected if there is damage to the articular disc, for example, dislocation of the disc and the condyle, resulting in excessive stretching of the TMJ ligaments [44]. This disorder, designated as DD or ID, can be defined as a malfunction and/or irregular location of the disc, often anteriorly [45,46]. It can be classified into four types (Table 1) [47,48].

TMJ DD can lead to TMJ clicks, brief pain and jaw movement limitations. Trauma and abnormal behaviors, such as tightness and bruxism are the most common causes of dislocation [49].

DD affects 35–42% of the worldwide population and up to 70% of patients with TMD [16,50]. The prevalence of this disorder is more common in young and middle-aged adults (20–50 years old) [51], with women being the most affected gender, in a ratio of 2:1 [52]. This disorder is not necessarily associated with the presence of symptoms or dysfunction [51].

### 3.2. Disc Structural Changes

The articular disc presents a biconcave shape. However, some morphological deformations may be found. These deformations may be classified as lengthened, biconvex, thickened, folded, and rounded [53,54,55,56].

DD is the main problem that leads to disc deformation. This deformation is mostly found on joints with DDwoR and begins with the thickening and enlargement of the posterior band of the articular disc [53,54,55]. Hasan and Abdelrahman supported these facts by proving that the risk of degenerative changes increased with the prevalence of an anterior displacement without the reduction of the disc, the anterior displacement being the most frequent type of occurrence. They also found a relationship between the articular structures and the anterior DD, where deterioration may occur in the disc and its ligaments if there is a posterior condylar position [57].

Disc degeneration leads to the formation of fibrous tissue and loss of normal TMJ disc morphology, such as thinning and perforation [53,58].

In conditions of closed-mouth position and teeth contact, if there is an extreme and long load, it could cause the thinning of the disc in the central band [59]. Moreover, cases of displacement could also lead to thinning of the posterior band [60].

Disc perforation could be related to osteoarthritis, rheumatic/inflammatory disease, or DD [61], normally with anterior DDwoR [62]. It usually ruptures in the bilaminar zone and the lateral part of the disc but depending on the type of DD it can perforate in different places [63]. The major problems related to perforation is the fact that the disc loses its functionality of lubrication due to interference with the synovial fluid, leading to increased friction between the articular structures of the TMJ and, consequently, resulting in hypertrophy [62,64].

A more recent study classified the deformities based on closed-mouth images and categorized them into folded, flattened, eyeglass, and amorphous. The authors concluded that disc perforation increases with the eyeglass and amorphous shapes, with age, abnormal joint space, and two or more structural changes in the condyle and fossa bones [64].

## 4. Temporomandibular Joint Disc Replacement Approaches

In the past, repair of a TMJ disc was almost non-existent due to technology constraints. Therefore, the only viable option was to replace it. The use of materials to reconstruct or replace the articular disc after TMJ discectomy began in the late 1970s and early 80s. The materials tested were silicone rubber (Dow-Corning, Midland, USA) [65] and Proplast-Teflon (Vitek, Inc., Houston, TX, USA) [66].

Silicone rubber became commercially available for medical purposes in 1962, with the first suggestion of the material being used for TMJ being made by Robinson M. in 1968. Silicone rubber was recurrently used for aesthetic surgery, joint replacements, and in oral and maxillofacial reconstructive surgery as well. The advantages of this material are the following: it is easily manipulated; it is a resilient material; and it easily adapts to the bone. Some surgeries were reported as being successful, while in others, patients developed severe symptoms, such as reactions to silicone particles and synovitis (inflammation of the synovial membrane), leading to the removal of the implants [67,68,69]. Since these implants proved to be inefficient in the long term, in 1989, Tucker, developed a study with the objective of temporarily applying silicone rubber implants in primates and removing them after 6 months of implantation. To this effect, after discectomy, a sheet of silastic, the silicone rubber implant was placed between the mandibular condyle and the glenoid fossa, and 6 months after surgery, an encapsulation of the implant by fibrous tissue was noticeable, with the implant effectively stimulating the formation of fibrous tissue and aiding the adhesions. Thus, with tissue formation, it would be possible to remove the implant and let the body produce the remaining structure by itself. However, it should be noted that for the application of silicone rubber implants, at least two surgeries are necessary, there is a high probability of an inflammatory response occurring in the first 3 months after application, and in the long term, this procedure can lead to the loss of the articular capsule [68,70].

Proplast-Teflon (polytetrafluoroethylene or PTFE) was also used as an implant in TMJ disc replacement [71]. This material was introduced in TMJ implants in 1976, with studies describing Teflon as more stable and with higher porosity when compared to silicone rubber, as the porosity plays an important role in cell adhesion [69,72]. The Proplast surface is placed close to the glenoid fossa to improve bone and fibrous tissue growth, given its porous structure which enhances implant stability. Teflon is positioned close to the condyle due to its smooth surface [73]. Sometimes this material is reinforced with vitreous carbon or aluminium oxide to smoothen it [74]. However, in 1990, the Food and Drug Administration (FDA) recommended removing these implants for cases where degenerative changes in the TMJ were observed [73]. The material quickly wore out and PTFE particles were shown to induce severe foreign body reactions resulting in granulomatous tissue and bone erosion [72].

In 2011, a review by Dimitroulis indicated the methylmethacrylate (MMA), a thermoplastic, for joint articular disc repair, offering an array of desirable characteristics: non-toxicity, low cost, compatibility, minor tissue inflammatory reactions, and high mechanical resistance [22,75]. However, other studies in literature, refer to the use of this material only for jaw reconstruction or cranioplasties [76,77,78].

There is also another method approved by the FDA, denominated Christensen (Ventura, CA, USA), in which patients with specific conditions and pathologies, resort to a prosthesis for total TMJ reconstruction. This method has existed for over 50 years and makes use of a cobalt-chromium (Co-Cr) prosthesis, in conjunction with a condylar prosthesis of a Co-Cr structure with a molded polymethylmethacrylate (PMMA) condylar head [23,79,80]. Although still in use up to this day, there are reported cases in which patients had to remove their implants due to the wear of the prosthesis and tissue necrosis where the prosthesis was implanted [81,82]. Thus, the various problems associated with both implants and prosthesis, such as bone resorption or inflammatory reactions, have led many researchers to discard this field of research [83] and attempted to reconstruct the TMJ disc with autogenous grafts, as temporalis muscle flat [84], auricular cartilage [85], full-thickness skin [86], dermal grafts [87] and dermal-fat grafts [88]. Despite some good reports, a critical review states that none of them satisfy the necessities for a successful replacement of the disc after discectomy [22]. The advent of TE has since gained more interest from the research community and is becoming one of the viable methods to repair the TMJ disc.

## 5. Approaches for Temporomandibular Joint Disc Substitution and Repair: Tissue Engineered Implants

TE is a very promising field for disc regeneration, especially if it is at an early stage [21]. Through this, solutions can be found for disc replacement or regeneration or alternatively to the replacement of structures in the TMJ [40]. Traditionally, the principal elements of TE are cells, stimuli, and scaffolds [16].

The first in vitro TE study of a TMJ disc was in 1991, where cells obtained from rabbit TMJ discs were combined with a collagen type I solution and, posteriorly, infiltrated into a porous collagen matrix and allowed to photopolymerize. Although the referred cells are composed of fibrocartilage, no fibrous matrix was found [89].

In 1994, synthetic materials, polylactic acid (PLA) and polyglycolic acid (PGA) fibers were used to form the shape of the disc, and chondrocytes (retrieved from bovine hyaline cartilage) were seeded onto the scaffold. After 1 week, the scaffolds were implanted subcutaneously into nude mice and results demonstrated evidence of hyaline cartilage formation and mechanical performance similar to the native donor cartilage [90]. Since then, different materials have been used for scaffold production. However, an ideal solution scaffold has yet to be found, since it is often associated with inflammatory responses and toxicity upon material degradation [21]. These issues can be overcome by TE, through the production of viable tissues that can renew themselves and display their normal function [40].

A suitable and successful TMJ disc TE must meet various criteria, as: being biodegradable and biocompatible, have a high load-bearing capacity and a suitable porosity and surface chemistry for cell differentiation. This mechanism is important due to the avascular structure of the disc and the fact that it is through the mechanical stimuli of the synovial fluid that cells receive glucose and oxygen [91].

To develop an optimized strategy for TMJ disc TE, the selection of the proper biomaterials is essential. It is then, with the selected biomaterials, that cells are incorporated and exposed to stimuli to build the desired extracellular matrix (ECM) microenvironment [92].

### 5.1. Biomaterials in Disc Regeneration

For a successful TE of the TMJ disc, the first big challenge is the selection of the appropriate biomaterial. Biomaterials can be characterized as materials for use in medical devices or for repairing biological tissues or organs. They can be divided into natural (animal or human origin), or synthetic materials [93,94]. They must meet several requirements to be applied in TE: biocompatibility with the host, to avoid an inflammatory response; biodegradability, to allow the material to be replaced by a suitable tissue; adequate permeability and architecture, such as porosity, to allow the transport and exchange of oxygen, nutrients, and waste; and appropriate mechanical properties relative to the tissue function [95,96].

**Figure 2:**
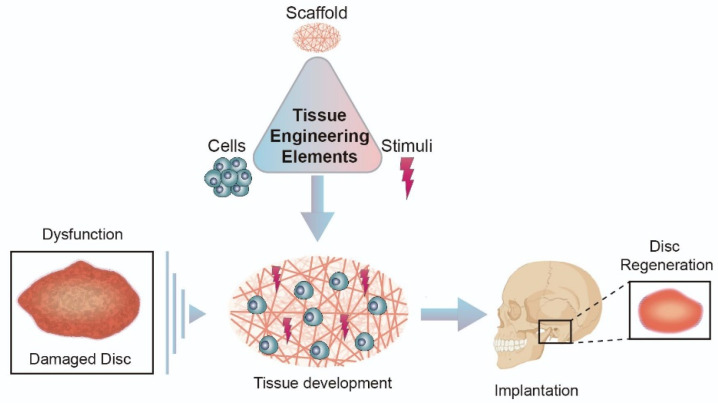
Tissue Engineering Strategy.

The current aim of biomaterials is to serve the necessary medical or surgical purposes to be safely implanted in the human body (Figure 2) [97]. Upon implantation, they are used to provide a biodegradable support structure, with desirable shape and integrity for an intended period of time, effectively providing functionality, support and attachment to the cells and give rise to the creation, and maturation of new tissue [93]. As for cartilage, TE must be able to create all the different structural organizations of the tissue to integrate the implant within the existing tissue [95].

#### 5.1.1. Natural Biomaterials

Natural biomaterials are derived from natural forms. They have a wide variety of applications in the biomedical field in the repair or replacement of biological tissues and organs. They present great biological requirements, such as biocompatibility, biodegradability, bioactivity, promote cell adhesion, proliferation, and differentiation, which are essential for tissue construction. Moreover, they present similar advantages to the biological macromolecules present in human tissues as they can be extracted from the shells of crustaceans (chitosan) or seaweeds (alginate). Despite these benefits, in certain situations, they present immunological reactions and some degree of variability, and there is the possibility of disease transmission. Another important fact is that natural materials can decompose at temperatures below their melting point [96,97,98]. This property can be a problem regarding material processability, which result in low mechanical properties and unstable degradation rates [99].

Few studies have demonstrated the potential of natural biomaterials (biopolymers) for TMJ disc TE. Chitosan presents excellent biocompatibility and provides adequate stimuli for cell proliferation for cartilage regeneration [99]. It was investigated the potential of two types of chitosan/alginate scaffolds for the differentiation of dental pulp stem cells. These scaffolds were produced by crosslinking calcium chloride (CaCl_2_) with or without glutaraldehyde in order to evaluate fibrocartilage production. The produced scaffolds presented interconnected porosity with pores with 100–300 µm. Results demonstrated the expression of fibrocartilage markers and the storage modules, and the elastic responses obtained proved to have identical values to the human native tissue. Despite these achievements, the anatomical shape of the disc, together with the biochemical distribution and alignment of its components, needs to be considered for the optimization of TE constructs [100].

The first in vivo TE study strategy consisted of the combination of fibrin/chitosan produced by freeze-drying with synovium-derived mesenchymal stem cells (MSCs). Results demonstrated that fibrin improved cell seeding efficiency and homogeneity, and after chondrogenic induction, it was possible to observe synthesized ECM structures related to cartilage. Despite these outcomes, after day 7 of cell seeding, the number of cells started to decrease [101].

Another research group demonstrated the potential of a collagen sponge scaffold seeded with autologous bone marrow MSCs, resulting in the formation of connective tissue in perforated TMJ discs of Japanese white rabbits, after only two weeks. The limitations of this work arise from the fact that rabbits only present rotation movements in the articulation, while humans also present gliding ones [102].

Alginate is a hydrogel used in cartilage TE and its use in the TMJ disc was evaluated. This component was seeded with TMJ disc cells and although cell migration into nodules was found in the first weeks of culture, histological results did not demonstrate collagen or GAG formation and even cellular population decrease in time [103].

#### 5.1.2. Synthetic Biomaterials

Compared to natural biomaterials, synthetic ones present several advantages, such as their availability and reproducibility as they are controllable and easy to process. For TE purposes, they can be modified according to the characteristics of the implant site to present adequate mechanical (stiffness, porosity and elasticity), physical and biochemical properties and degradation rate. The major issue with these materials is the structural difference compared to native tissues, which may lead to a negative effect on biocompatibility [98,104,105]. Even more, it is associated with limited cell adhesion sites and homogeneous cell proliferation, which compromises tissue synthesis. Still, some biomaterials have high biocompatibility and incorporate well into the human body [8].

Several studies have shown the potential of synthetic biomaterials in the regeneration of the TMJ disc. PLA, due to its slow degradation time, has been studied with the incorporation of adipose stem cells. Mäenpää et al. report the first study regarding this type of cells for TE of the TMJ disc, combined with nonwoven PLA discs. The expression of aggrecan and collagen type I and II increased in a chondrogenic medium, but the differentiation degree of the cells was lower when compared to cells derived by the TMJ disc [106].

Fabrication of non-absorbable scaffolds was also carried out by a group of researchers, in which four types of scaffolds were produced: polyamide (PA) monofilaments and expanded polytetrafluoroethylene (ePTFE) monofilaments. PGA monofilaments and natural bone mineral blocks were used as control. Cells were taken from the TMJ disc and articular eminence (both from human and porcine tissues) and implanted on the referred scaffolds. Results demonstrated cell attachment to all of the produced scaffolds, independently of their nature. Although the implanted cells were fibrocartilage, the production of collagen type II was found, not resembling the properties of the TMJ disc which is mainly composed of collagen type I [107].

A study in the literature showed that higher TMJ disc cell seeding on PGA structures, results, in an increased amount of matrix production. Despite this, there was a decrease in cell population over the culture period and the higher seeding density scaffolds revealed a 50% decrease in volume [108]. PGA, which presents a rapid degradation rate, and non-woven poly-L-lactic acid (PLLA) meshes with a slower degradation rate were manufactured. These latter constructs had the capability of maintaining their volume for 6 weeks, when compared to PGA, but presented low mechanical capacity. Transforming growth factor-beta 1 (TGF- β1) incorporation generated a high quantity of cells, collagen, and GAGs. These two studies demonstrate that the reduction of volume found may be due to the rapid degradation of PGA [109].

Hagandora et al. tested the use of poly (glycerol sebacate) (PGS) with the incorporation of fibrochondrocytes. Different biochemical and biomechanical properties were obtained due to different cell seeding densities and culture times, where the longest culture time and higher cell seeding resulted in higher ECM production. Nevertheless, a non-homogeneous distribution of cells and matrix was found [110].

Recently, poly(ε)-caprolactone (PCL) scaffolds have been studied due to their slow degradation. Legemate, Tarafder, Jun, and Lee produced PCL scaffolds by 3D printing, where fiber orientation represented the collagen network. Spatiotemporal delivery of connective tissue growth factor (CTGF) and transforming growth factor beta 3 (TGF- β3) were incorporated and the different regions of the disc were mechanically evaluated (anterior, posterior, and intermediary). The final constructs not only present region-dependent MSC differentiation but also the viscoelastic properties are region-dependent. Authors state that an in vivo and long-term scaffold degradation tests needs to be performed to validate this implant [111].

Our group studied the combination of polyethylene glycol diacrylate (PEGDA) with 3D-printed PCL scaffolds. We hypothesized that the PCL would confer the necessary mechanical performance, while the PEGDA hydrogel would help in lubrification. Results demonstrated that the hydrogel as a core in the scaffold mimics the mechanical properties of the native tissue, although in vitro and in vivo studies are essential to validate this proposal [112].

Although all the points before mentioned are valid, biomaterials after manipulation are not able to properly mimic the necessary microenvironment of the tissue [113], which is why the use of composite materials (natural combined with a synthetic material) might effectively help to overcome this problem [105].

## 6. Forefront Approaches for Temporomandibular Disc Replacement: Native Decellularized Extracellular Matrices

**Figure 3:**
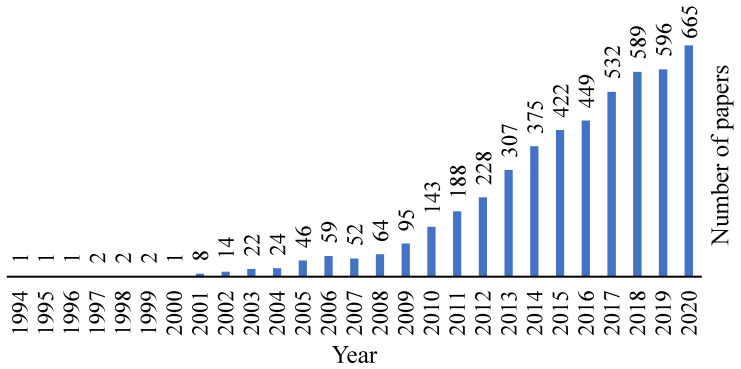
Evolution of the decellularization area over the years. Retrieved from PubMed.org with the research designation “decellularization”, where 4140 results were found.

Decellularized tissues are a well-known matter in the biomedical field and by analysing Figure 3, it is possible to observe that this area is being increasingly explored and evolving over the years.

Cartilage has a low regeneration capacity and therefore, different substitutes have been the focus of research in order to repair cartilage defects. Cartilage matrix can be collected from different sources, but access to allogeneic or autologous donor tissue is restricted, so the interest in using xenogeneic tissues for cartilage constructions has been increasing, where the TE field can offer a positive alternative [114,115]. Regardless, for these tissue types, decellularization and sterilization methods are required with the aim of removing the immunogenic components that lead to infection and disease transmission [114,116].

The decellularized extracellular matrix (dECM) has immense potential to serve as a beneficial material for tissue damage repair as it preserves the native environment by providing cells with the necessary elements, such as support and biochemical components, that are needed to provide their proliferation and differentiation. ECM organization and compounds differ from tissue to tissue [113], but in terms of cartilage, the two major components are collagen and proteoglycans, which include bioactive factors, such as growth factors, integrins, and functional peptides. The main benefits of using dECM are related to its ability to preserve native tissue growth factors (e.g., transforming growth factor beta (TGF-β), fibroblast growth factor (FGF) and insulin-like growth factor (IGF) for cartilage tissue), unlimited access to obtain ECM and the relationship between cost and effectiveness [115]. Still, there are problems associated with it and that may create undesired responses. The remaining cell contents, heterogeneous cell distribution, and the difficulty of obtaining an intact ECM are some of the cautions to pay attention to [94].

Choosing the right animal model for any given tissue is a critical step, and the decellularization method depends on the tissue choice [116]. Decellularization methods can be divided into (i) chemical agents, such as acids and bases, detergents, hypotonic and hypertonic solutions, and solvents, (ii) biological agents, such as enzymes and chelating agents; and (iii) physical agents, such as freeze-thaw, force and pressure, electroporation and sonication [25,117].

**Figure 4:**
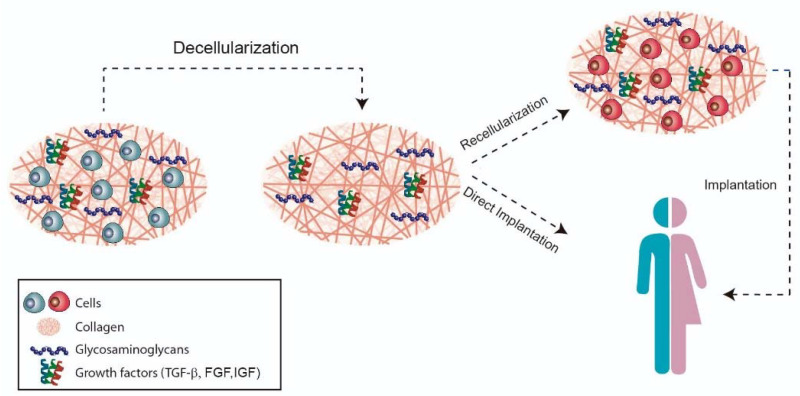
Decellularization strategy.

The aim of decellularization (Figure 4) is to preserve the organic and mechanical properties, such as the architecture of the collagen network of the tissue, as the immunogenic components are removed to allow cell adhesion and proliferation. After obtaining the xenogeneic scaffold, there are two possible ways of application: direct implantation or cell culture in the decellularized scaffold [114,116].

Brown et al. reports the first use of powdered porcine urinary bladder ECM encapsulated within sheets of the same material for TMJ disc after discectomy [118]. To improve this work, an in vivo study was performed in a canine model and a morphological and biomechanical characterization was performed. Results demonstrated that the implantation of an acellular scaffold supported tissue formation similar to the native one. However, this implant presented rapid degradation and histological analysis of the condyle and temporal fossa needs to be performed [119].

More decellularization protocols are still needed as cells are trapped in a dense ECM. Because of this, upon the decellularization processes, GAGs may be destroyed and tissue thickness may decrease affecting their biomechanical properties [115].

Few studies have focused on the best method for the effective decellularization of the TMJ disc. The first study investigated three different decellularization methods in porcine disc: 1% (*w*/*v*) sodium dodecyl sulfate (SDS), 1% Triton-X, and 1:4 (vol%) acetone/ethanol. It was found that SDS was more effective in removing cellular content, maintaining the modulus values and energy dissipation capability according to that found in the native disc, although showing some collagen fibers compression [116]. This study was later used as a basis for other studies related to disc decellularization. Juran et al. tested 1% SDS for porcine discs decellularization, where the same result was obtained regarding the collagen fibers. However, after lyophilization and rehydration, the fibers were swollen and resembled the collagen network of the native disc more. The efficacy of laser micropatterning for producing artificial porosity was also tested. Holes of 120 µm were laser drilled on the lyophilized disc, resulting in cell remodeling over a 21-day culture time. Despite the authors referring to the fact that the mechanical properties were maintained, some differences were still found between the native and the laser micropatterned in the hydraulic permeability coefficient (1.79 × 10^−16^ ± 0.04 × 10^−16^ vs 1.06 × 10^−16^ ± 0.10 × 10^−16^ m4/Ns) and compressive modulus (1.65 ± 0.24 vs 2.20 ± 0.24 MPa) [91].

To be able to focus on the decellularization of the TMJ disc along with its retrodiscal tissue, new decellularization agents need to be investigated. In addition, if porcine discs are to be considered, one needs to pay attention to the fact that this particular retrodiscal tissue has more lipid content than the human tissue. A valid scaffold approach for this issue was investigated and a proposition for the combination of SDS and chloroform/methanol was made to effectively decellularize the disc-retrodiscal tissue complex [120].

Another study concluded that an agitation method for decellularization combined with 0.1% of SDS preserves the ECM while minimizing the risk of residual SDS. Laser micro ablation was also evaluated to understand whether it should be performed before or after decellularization. It was concluded that it should be done afterward, since it presents smaller and more uniform holes, being related to a lesser alteration of the biomechanical properties [121].

More recently, Liang et al. developed an injectable hydrogel based on decellularized porcine TMJ discs. A combination of physical (freeze-thaw), chemical (1% Triton X-100 and hypotonic Tris–hydrochloric acid buffer (Tris-HCL)) and enzymatic (trypsin and nucleases) methods were used, where a significant reduction of sulfated GAGs was found. The hydrogel was combined with encapsulated chondrocytes and injected into a mouse, but a small inflammation was observed within 7 days [122].

Despite the discoveries so far, there are still problems that need some attention. As described in this review, investigations related to the most effective method of decellularizing the disc are scarce, thus our research group is currently extensively studying other methods of decellularization of the TMJ disc, such as chemical, physical and enzymatic, in order to find the most effective strategy. In parallel, as part of a project also developed by our group, named -bio-discus, a biomechanical model of a sheep skull is being built. The purpose of this model is to perform mechanical tests to potential discs to be applied in the replacement of the native disc, since it simulates the forces exerted on the TMJ disc during the masticatory movements. The use of this biomechanical model will drastically reduce animal testing and, at the same time, increase the potential success of the developed implant.

In Table 2, there is a summary of the TE approaches, as well as the best methods of decellularization proposed by different authors for the TMJ disc, which were presented throughout this review

## 7. Conclusion and Future Strategies

The temporomandibular disc is a complex structure, with specific collagen and GAG distribution. It is a fibrocartilaginous disc with no vascularization and remodeling capacities. This combined with its dynamic properties in the normal function of the TMJ, makes this tissue to be highly predisposed to suffer pathologies. This review was able to present past and current replacement strategies for the TMJ disc and demonstrated the short- and long-term complications associated with each of them.

The TE field has actively contributed to the possibility of bringing new outcomes to the search for a new and long-lasting tissue that effectively substitute/regenerates the disc. Different materials were analyzed and their advantages and disadvantages were highlighted. Still, the production of a suitable material that best imitates the native properties of the disc, such as mechanical, physical, and biological, with no negative reactions, has not been achieved.

Our group believes that studies involving decellularized xenogeneic tissues may be the next step in the development of a native-equivalent disc. Since, in a simplified way, it is possible to obtain the necessary characteristics (physical and biochemical) to successfully obtain one engineered-disc for the treatment of disc pathologies.

## Figures and Tables

**Table 1:** Different types of Disc Displacement (DD).

Disc Displacement Types		Characterization
Disc Displacement with Reduction (DDwR)		The articular disc is dislocated but able to return to its initial position with condyle translation
Disc Displacement without Reduction (DDwoR)	With limited opening (DDwoRwLO)	The articular disc is locked, not being able to return to its initial position. Presents a restricted mouth opening
	Without limited opening (DDwoRwoLO)	The articular disc is locked, not being able to return to its initial position. Does not present a restricted mouth opening

**Table 2:** Natural and synthetic materials used for tissue engineering of the TMJ disc.

Author	Material/Tissue	Fabrication/Decellularization Method	Cells/Growth Factors	Benefits	Limitations
Tissue Engineering: Natural Materials					
Thomas et al. [89]	Collagen	Photopolymerisation	Rabbit TMJ disc cells	Growth of a tissue analog in vitro	No fibrous matrix formation
Almarza & Athanasiou [103]	Alginate	Crosslinking with CaCl_2_	Hogs TMJ disc cells	Cell migration into nodules in the first weeks of culture	No collagen or GAG formation; decrease in the cell population
Wu et al. [101]	Combination of fibrin and chitosan	Freeze-drying	Synovium derived MSCs	Fibrin improved cell seeding efficiency; ECM synthesis	Number of cells started to decrease after day 7 of cell seeding
Kobayashi et al. [102]	Collagen	Freeze-drying and thermal crosslinking	Bone marrow MSCs	Connective tissue formation	In vivo implantation in rabbits, that only present TMJ rotation movements
Bousnaki et al. [100]	Combination of chitosan and alginate	Crosslinking with CaCl_2_	Dental pulp stem cells	Fibrocartilage markers expression; adequate mechanical properties	TMJ disc shape and biochemical components were not evaluated
Tissue Engineering: Synthetic Materials					
Puelacher et al. [90]	Combination of PLA and PGA fibers	Spraying fixing technique	Calves chondrocytes	Adequate mechanical properties	Hyaline cartilage formation
Springer et al. [107]	PA, ePTFE and PGA monofilaments	Plaiting	Human and porcine TMJ disc and articular eminence cells	Cell attachment to all scaffolds, independently of the cells	Collagen type II production
Almarza & Athanasiou [108]	PGA mesh	Not specified (purchased)	Hogs TMJ disc cells	Higher seeding cells results in increased matrix production	Decrease in cell population over the culture period; scaffolds decrease 50% in volume
Allen & Athanasiou [109]	PLLA mesh	Not specified (purchased)	Hogs TMJ disc cells and TGF- β1	Scaffold volume maintained for 6 weeks; Growth factor incorporation yielded cells, collagen and GAG	Low mechanical properties
Mäenpää et al. [106]	PLA	Melt-spun	Adipose MSCs	Expression of aggrecan and collagen type I and II	Low degree of cells differentiation
Hagandora et al. [110]	PGS sheets	Particulate leaching	Goat TMJ disc cells	High ECM production	Non-homogeneous distribution of cells and matrix
Legemate et al. [111]	PCL	Fused deposition modelling	Bone marrow MSCs, CTGF and TGF- β3	MSCs differentiation and viscoelastic properties are region-dependent	To validate this proposal in vivo and long-term scaffold degradation studies are required
Moura et al. [112]	Combination of PCL and PEGDA	Combination of fused deposition modelling and photopolymerisation	___	PEGDA as a hydrogel core presents adequate mechanical properties	To validate this proposal in vitro and in vivo studies are required
Decellularization					
Brown et al. [118,119]	Urinary bladder matrix (turned into powder)	0.1% peracetic acid/4% ethanol	___	*In vivo* test led to tissue formation	Rapid degradation; Lacks histological analysis of the bony structures
Lumpkins et al. [116]	Porcine TMJ disc	1% (*w*/*v*) SDS	___	Maintained mechanical properties; Cell removal	Collagen fiber compaction; no biochemical quantification
Juran et al. [91]	Porcine TMJ disc	1% (*w*/*v*) SDS, lyophilization, rehydration and micropatterning	Umbilical cord MSCs	Cell removal; cell integration and remodelling	no biochemical quantification; Low mechanical properties
Matuska et al. [120]	Porcine TMJ disc	0.1% (*w*/*v*) SDS and 2:1 solution of chloroform/methanol	Umbilical cord MSCs	Cell and lipid removal; No citotoxicity	Mechanical properties of the reagents were only assessed separately; no biochemical quantification
Matuska & McFetridge [121]	Porcine TMJ disc	0.1% (*w*/*v*) SDS and micropatterning	___	Cell removal; Minimal collagen lost	No GAG quantification; in vitro studies were not performed to evaluate the micropatterning
Liang et al. [122]	Porcine TMJ disc (solubilised and processed into hydrogel)	Freeze-thaw, 1% Triton X-100, Tris–HCL, trypsin and nucleases	Rabbit chondrocytes	Cell removal; Good injectability and degrability; hydrogel with nanofibrous structure	Sulfated GAG reduction; in vivo inflammation
